# A Cardiovascular Mathematical Model of Graded Head-Up Tilt

**DOI:** 10.1371/journal.pone.0077357

**Published:** 2013-10-29

**Authors:** Einly Lim, Gregory S. H. Chan, Socrates Dokos, Siew C. Ng, Lydia A. Latif, Stijn Vandenberghe, Mohan Karunanithi, Nigel H. Lovell

**Affiliations:** 1 Department of Biomedical Engineering, Faculty of Engineering, Kuala Lumpur, Malaysia; 2 Graduate School of Biomedical Engineering, University of New South Wales, Sydney, Australia; 3 Department of Rehabilitation Medicine, Faculty of Medicine, University of Malaya, Kuala Lumpur, Malaysia; 4 ARTORG Cardiovascular Engineering, University of Bern, Murtenstrasse, Bern, Switzerland; 5 The Australian EHealth Research Centre, CSIRO, Herston, Queensland, Australia; University of Adelaide, Australia

## Abstract

A lumped parameter model of the cardiovascular system has been developed and optimized using experimental data obtained from 13 healthy subjects during graded head-up tilt (HUT) from the supine position to 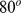. The model includes descriptions of the left and right heart, direct ventricular interaction through the septum and pericardium, the systemic and pulmonary circulations, nonlinear pressure volume relationship of the lower body compartment, arterial and cardiopulmonary baroreceptors, as well as autoregulatory mechanisms. A number of important features, including the separate effects of arterial and cardiopulmonary baroreflexes, and autoregulation in the lower body, as well as diastolic ventricular interaction through the pericardium have been included and tested for their significance. Furthermore, the individual effect of parameter associated with heart failure, including LV and RV contractility, baseline systemic vascular resistance, pulmonary vascular resistance, total blood volume, LV diastolic stiffness and reflex gain on HUT response have also been investigated. Our fitted model compares favorably with our experimental measurements and published literature at a range of tilt angles, in terms of both global and regional hemodynamic variables. Compared to the normal condition, a simulated congestive heart failure condition produced a blunted response to HUT with regards to the percentage changes in cardiac output, stroke volume, end diastolic volume and effector response (i.e., heart contractility, venous unstressed volume, systemic vascular resistance and heart rate) with progressive tilting.

## Introduction

Cardiovascular response to upright posture has been widely studied through numerous experiments and mathematical models. The primary hemodynamic effect of upright posture is the redistribution of blood volume to the lower portion of the body, depending on regional variations in the compliance, hydrostatic changes in the pressure, vasomotor activity and transmural pressure applied to the vascular walls by the surrounding tissue [1]. This is accompanied by a rapid fall in the central venous pressure, marked reduction in the ventricular filling pressure and subsequently a decrease in the stroke volume [2]. A few mechanisms have been suggested to counteract the negative effects of upright posture and therefore maintaining circulatory stability, including rapid adaptation of the smooth muscle of arterial and venous vessels to changes in their diameters or perfusion pressures; autonomic reflexes, particularly arterial and cardiopulmonary (CP) baroreceptors which induce an increase in heart rate, peripheral vascular resistance, venous tone and heart contractility; the ‘skeletal muscle pump’ of the lower body; and neurohormones [3].

Various mathematical models have been developed to investigate the interaction among the complex mechanisms involved during postural changes, including active standing [4]–[7], head-up tilt (HUT) [8], [9] and lower body negative pressure [8], [10]. Melchior *et al.* [11] presented a comprehensive review of the important features involved during orthostatic stress, for example nonlinear blood vessel compliance, fluid filtration, role of extravascular pressures, as well as both reflex control and autoregulation mechanisms. The inclusion of these features in the model depends on the specific phenomenon the model intended to study. For example, the skeletal muscle pump mechanism which helps to drive venous blood back to the right atrium is proposed to be an important mechanism during active standing [3]. On the other hand, Heusden *et al.* [9] proposed that viscoelastic stress relaxation of the systemic veins and direct effect of gravity on the pulmonary circulation play an important role in reproducing the transient seen during HUT. Reflex control mechanisms, especially the arterial baroreflex and cardiopulmonary (CP) reflexes, were included in most models to accurately reproduce the response to orthostatic stress. Olufsen *et al.* [5], [6] included cerebral autoregulation in their model to capture the transient response in pressure and cerebral blood flow velocity which acts to compensate for hypotension during standing.

Although numerous computational models have been developed to investigate the cardiovascular response to upright posture [4]–[10], none of these studies have evaluated the effects of HUT in heart failure subjects. To date, we are only aware of one study by Ha *et al.* [4] which simulated the effects of posture and ventricular function using an integrative model of the ovine cardiovascular system. The main limitation of the study was that the simulated left ventricular (LV) end diastolic pressure was too low to reasonably model the decompensated heart failure state, and therefore was not able to highlight the differences between a healthy and heart failure patient with regards to their response to upright posture. Furthermore, although they attempted to validate their baseline supine hemodynamic variables (i.e. heart rate, stroke volume, cardiac output, mean aortic root and pulmonary arterial pressure, LV end systolic and end diastolic pressures, as well as ejection fraction) against experimental findings, no effort was carried out to validate their simulated results to upright posture, which included transient response of the mean arterial and pulmonary arterial pressure, heart rate, cardiac output and LV/RV pressure volume loops upon standing.

The major motivation for the present study was to develop a cardiovascular system (CVS) model that is able to accurately reproduce the steady state hemodynamic responses during HUT at various tilt angles (from the supine position to 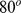), under both healthy and congestive heart failure conditions. The CVS model consists of both the systemic and pulmonary circulations, and was divided into a few parallel branches to account for the differences in heights of each compartment from the hydrostatic indifference point (HIP) located at the heart level, as well as different impacts of regulatory mechanisms on these compartments. A number of important features including the separate effects of arterial and CP reflexes, the autoregulation mechanism in the lower body, as well as diastolic ventricular interaction through the pericardium have been included. A data set of cardiovascular measurements during supine baseline posture and various degrees of HUT in 13 healthy subjects, as well as data from the literature were used to tune the model parameters.

## Materials and Methods

### Experimental Description

#### Subjects

The experimental data used for model validation were collected from 13 healthy subjects (12 males and 1 female, aged 18–44 years, mean age 30 years). Prior to the experiment, subjects were requested to provide information about their physical condition and none reported any history of cardiovascular or respiratory disease. Written informed consent was obtained from all participants, and the study was approved by the Human Research Ethics Advisory (HREA) Panel of the University of New South Wales.

#### Measurement devices and systems

Doppler flow velocity was measured at the ascending aorta using the USCOM ultrasonic cardiac output monitor (USCOM, Sydney, Australia). The USCOM device uses a handheld piezoelectric 2.2 MHz continuous wave (CW) Doppler ultrasound probe to insonate the aortic flow. As recommended by the manufacturer, the probe could be placed either at the suprasternal notch or at the supraclavicular fossa area (lateral to the sternocleidomastoid muscle) for the measurement of aortic flow. During each subject trial, measurement attempts were made at both positions, and the probe position that consistently produced high quality images at all the tilt angles was chosen as the preferred probe position. The USCOM device employed in the current study was a modified version that allowed the digital images of aortic flow velocity profiles to be downloaded to a computer for further processing. ECG was acquired from the lead I configuration and amplified with a bioamplifier (ADInstruments, Sydney, Australia). The signals were recorded and digitized at a sampling rate of 1000 Hz using the Powerlab data acquisition system (ADInstruments, Sydney, Australia). BP measurements were obtained using a clinically approved oscillometric BP device (Colin Co., Japan) from a cuff placed around the left arm over the brachial artery.

#### Measurement protocol

The subjects were advised not to eat for at least 2 hours prior to the study, with any meal to be free of alcohol and caffeine beverages. The subjects were also asked not to undertake any intensive exercise within 12 hours before the study. All measurements were made in a quiet dimly lit room at an ambient temperature of approximately 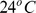. The subject initially rested in a supine position on the tilt table for a period of 20 minutes.

The subject's feet were supported by a footboard, and straps were applied at the levels of waist and knees to stabilize the body during HUT. Measurements were made at each of the following tilt angles in incremental order: supine (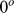), 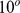, 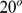, 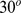, 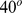, 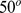, 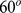 and 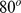. At each tilt angle, ECG was recorded for a period of 15 s, immediately followed by 15 s of aortic flow measurement and BP measurement. After each tilt, a 1.5 min adaptation period allowed the measured cardiovascular variables to settle to a stable level, which generally takes up to 30 s [12]. Measurements were made with the subject breathing spontaneously. BP measurements were acquired with the subject's forearms supported by armrests maintained at close to the heart level.

#### Signal processing and data analysis

All signal processing and data analysis were performed in Matlab (the MathWorks Inc., Natick, USA). The R-wave peaks were detected from the ECG signal using a set of automatic programming routines involving lowpass filtering, differentiation, and threshold peak detection. RR was computed as the time interval between successive R-wave peaks and heart rate was calculated accordingly. A program was written to track the instantaneous peak aortic flow velocity values from the spatial velocity spectra based on the threshold method [13]. The aortic velocity time integral (VTI) was computed as the area under flow velocity curve during the systolic ejection phase, and the percentage change in aortic VTI was taken as an estimate of the percentage change in stroke volume. The HR and aortic VTI measurements of a subject at a given tilt angle were averaged over the 15 s recording period.

### Model Description

**Figure 1:**
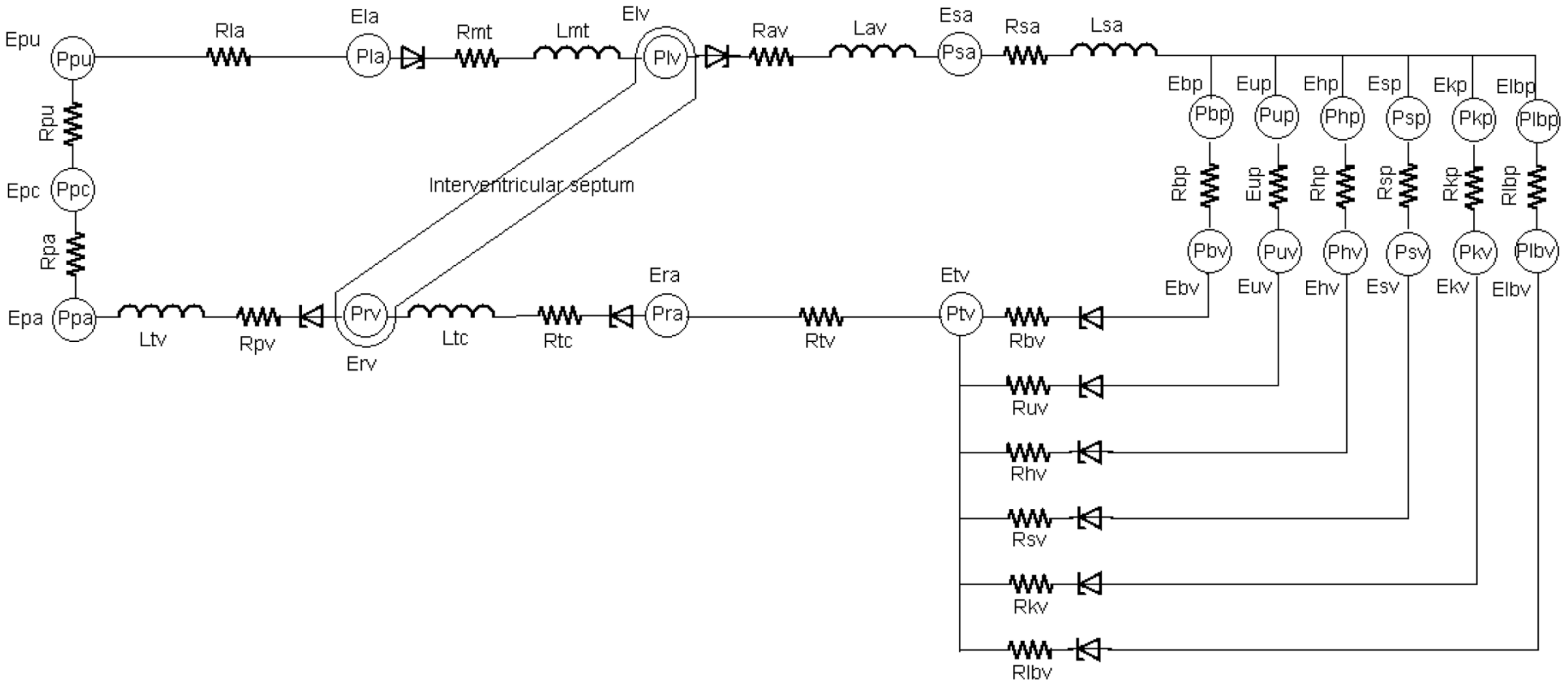
Electrical equivalent circuit analogue of the cardiovascular system (CVS) model. 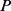
, pressures; 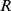, resistances; 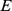, elastances ( = 1/compliances); 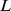, inertances. The CVS system includes (1) heart chambers consisting of the left and right atria, as well as the left and right ventricles; (2) pulmonary circulation consisting of the series arrangement of the arteries, capillaries and venous circulation; and (3) systemic circulation consisting of the large arteries and distinguishing between the peripheral and venous circulation of six parallel vascular districts: cerebral, upper body, coronary, splanchnic, renal, and lower body. Abbreviations: la, left atrium; lv, left ventricle, sa, large arteries; bp & bv, cerebral arteries & veins; up & uv, upper body arteries and veins; hp & hv, coronary arteries and veins; sp & sv, splanchnic arteries and veins; kp & kv, renal arteries and veins; lbp & lbv, lower body arteries and veins; tv, thoracic veins; ra, right atrium; rv, right ventricle; pa, pulmonary arteries; pc, pulmonary capillaries; pu, pulmonary veins.

An electrical equivalent circuit analogue of the lumped parameter CVS model is illustrated in Fig. 1. The model consists of the left and right sides of the heart, both the pulmonary and systemic circulations, as well as the reflex control system, each of which is described in the following sections.

#### Heart chambers

The structure of our heart model was adopted from [14], [15], which includes the left and right atria, left and right ventricles, as well as formulations for the direct ventricular interaction through the septum and pericardium. Luminal volumes of the left and right ventricles were enclosed by the left and right ventricular free-walls and separated by the interventricular septum. Movement of the cardiac muscles was constrained by the fluid in the pericardial space. Each of the four heart chambers and the septum was modelled as a volume surrounded by an elastic material obeying an assumed pressure volume (PV) relationship which varies from exponential during diastole to linear during systole, depending on the time varying elastance function, 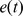. The transmural pressure across the pericardial wall was described by a passive PV relationship similar to the end diastolic PV relationship of the heart chambers. On the other hand, heart valves in our model were modelled using a resistance, 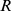, in series with an inertance, 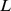, taking into account the significant change in flow velocity through the heart valves. The valves allow flow only when the pressure gradient across them is positive. When this gradient reverses, blood flow decelerates and finally closes the valve. Detailed descriptions of the heart model and their corresponding parameter values can be obtained from [15].

#### Circulation system

The circulatory model includes both the systemic and the pulmonary circulations. The systemic circulation was further divided into six parallel vascular districts: cerebral, upper body, coronary, splanchnic, renal, and lower body which includes the leg and pelvic region. The distribution of the systemic circulatory compartments was made to account for the difference in heights of each compartment from the HIP located at the heart level [9], as well as different impacts of regulatory mechanisms on these compartments [16]. Flow and volume distributions in each compartment in the supine position is given in Table 1 [17]–[19].

**Table 1:** Flow and volume distributions in each of the six vascular compartments during supine position, as well as their respective distances from the hydrostatic indifference point (HIP).

Compartment	Volume (%)	Flow (%)	Distance from HIP (cm)
Cerebral	7.3	14.9	−38
Upper body	11.3	9.8	−15
Coronary	2.8	5.0	0
Splanchnic	33.5	31.0	12
Renal	8.5	24.5	17
Lower body	17.4	14.8	65

Volume conservation laws were used to model the net change of volume for each compartment, given by
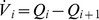
(1)where 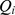 is the inflow while 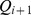 is the outflow.

Gravitational effects during HUT were taken into account in all compartments by introducing a hydrostatic pressure, 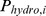, to each compartment according to their distance from the HIP [9]:
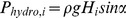
(2)where 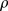 denotes blood density, 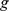 denotes gravity acceleration, 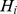 denotes distance between the 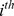 compartment and the HIP (given in Table 1), 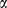 denotes the angle of tilting from the supine position.

The pressure in each compartment (except for the lower body compartment), 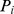, depends on the volume of the compartment, 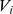, compliance of the compartment, 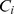, unstressed volume of the compartment, 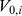, as well as its respective distance from the HIP, through the linear PV relationship:
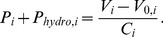
(3)


On the other hand, the nonlinear PV relationship proposed by Melchior *et al.* [10] was adopted for the lower body compartment, as transmural pressure of the lower body compartment increases greatly at high tilt angles:
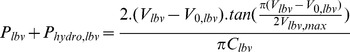
(4)where 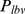 & 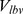 denotes pressure and volume of the lower body compartment, 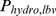 denotes hydrostatic pressure of the lower body compartment, 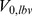 denotes unstressed volume of the lower body compartment, 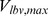 denotes maximum volume of the lower body compartment (700 mL), and 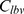 denotes compliance of the lower body compartment at low blood volume.

Flow between two compartments, 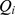, depends on their pressure difference, 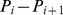, and the resistance between them, 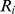:
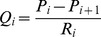
(5)where 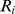
's were assumed to be constants for the pulmonary circulation and the systemic arteries. Venous valves have been included in each compartment of the systemic circulation to prevent retrograde flow, by the insertion of an ideal diode downstream of the venous compliance [16].

Parameter values for the circulatory model were either based on the literature [16], [20] or estimated, using supine steady state hemodynamic parameters as starting point.

Total resistance of the systemic circulation was assumed to be 1.06 mmHg.s/mL, while that of the pulmonary circulation was 0.13 mmHg.s/mL [20]. Resistance of each individual compartment was either obtained from the literature [16], [20] or estimated according to their flow distribution (parameter values listed in Table 2).

**Table 2:** Values of the model parameters characterizing the circulation and regulatory system.

Compliance (mL/mmHg)	Unstressed volume (mL)	Resistance (mmHg.s/mL)	Regulatory model	Regulatory model
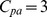	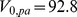	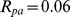	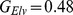	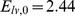 mHg/mL
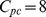	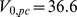	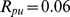	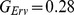	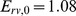 mHg/mL
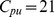	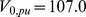	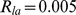	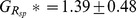	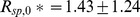 mmHg.s/mL
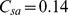	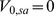	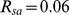	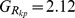	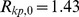 mmHg.s/mL
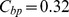	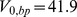	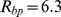	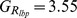	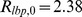 mmHg.s/mL
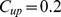	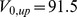	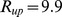	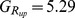	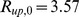 mmHg.s/mL
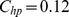	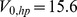		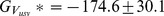	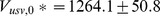 L
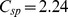	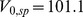	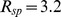	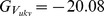	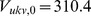 mL
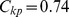	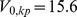		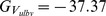	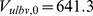 mL
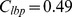	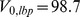	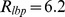	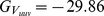	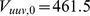 mL
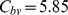	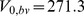	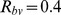	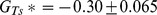	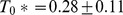 s
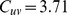	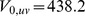	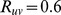	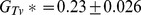	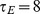 s
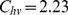	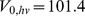	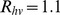	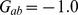	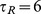 s
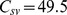	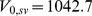	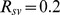	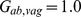	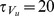 s
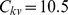	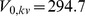	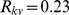	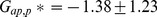	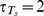 s
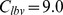	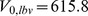	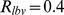	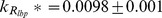	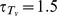 s
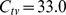	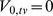	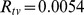		

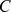
: compliances; 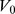: unstressed volumes; 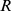: resistances; 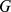: gain factors; 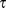: time constants of the controlled parameters; Subscript 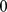: values of the controlled parameters when both vagal and sympathetic activities are zero. (*) These values (mean 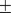 standard deviation) were determined through least squares optimization on measurements obtained from the 13 healthy subjects.

On the other hand, total compliance of the systemic circulation was assumed to be 118 mL/mmHg, while that of the pulmonary circulation was 32 mL/mmHg [20]. Compliances and unstressed volumes of the different compartments were either obtained from the literature [16], [18]–[20] or estimated according to their volume distribution (parameter values listed in Table 2).

#### Regulatory mechanisms

**Figure 2:**
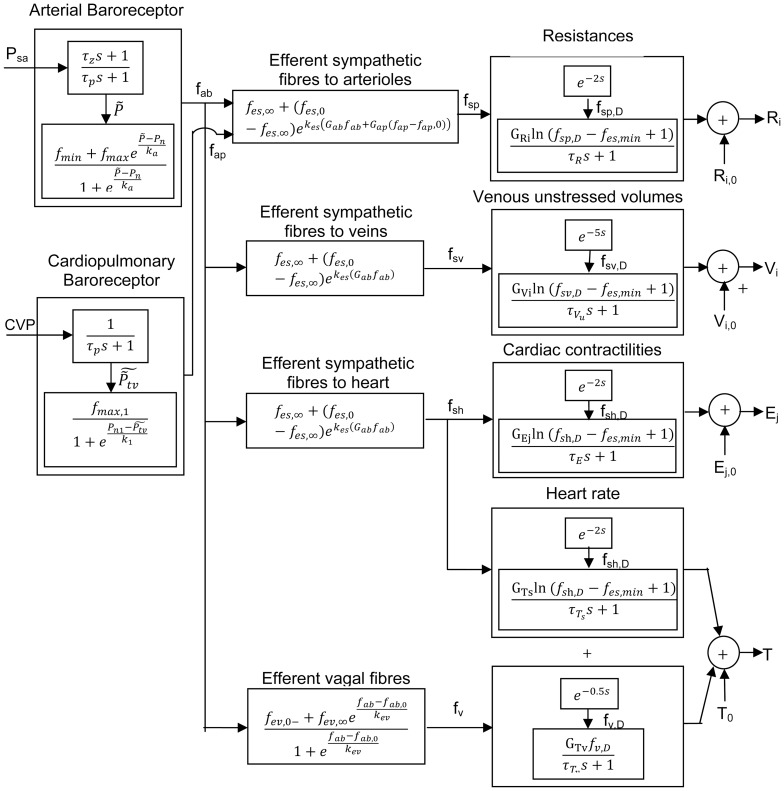
Block diagram of the complete reflex model. 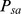
: systemic arterial pressure; 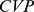: central venous pressure; 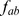 & 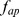: afferent activities from the arterial and cardiopulmonary receptors respectively; 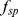, 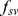 & 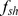: efferent sympathetic activities directed to the systemic resistances, veins and heart respectively; 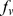: efferent vagal activity; 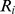 & 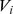: systemic resistances and venous unstressed volumes (i  =  splanchnic,renal,upper body,lower body); 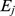: cardiac contractilities (j  =  left ventricle, right ventricle); 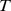: heart period.

The structure of the regulatory mechanisms model was adopted from [20], [21], which includes the arterial and the cardiopulmonary (CP) baroreceptor components. The model consists of the afferent pathways from the arterial and CP baroreceptors, the efferent sympathetic and parasympathetic activities, and the responses of several distinct effectors, including the heart contractilities, peripheral resistances, unstressed volumes and heart rate (Fig. 2). Since the arterial baroreceptors in the aortic arch and the carotid sinus are located above the heart level, the input pressure to the afferent pathway is the aortic pressure at heart level adjusted with a hydrostatic column (assumed to be 16 cm above the heart level), 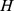. Therefore, the afferent arterial baroreflex pathway was modified as follows:
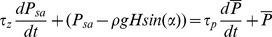
(6)where 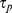 and 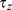 are the time constants for the real pole and the real zero, respectively, in the linear dynamic block of the baroreflex pathway, 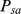 is the large arterial pressure, 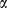 denotes the angle of tilting from the supine position, and 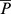 is the output variable of the linear dynamic block [20]. On the other hand, the afferent CP baroreflex pathway is modeled as a function of the central venous pressure.

In order to account for the greater increase in the vascular resistance of the leg as compared to the arm (2∶1) during HUT [22], we modelled the lower body resistance, 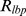, using the following equation to include the effect of the myogenic response:
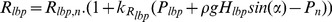
(7)where 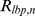 depends on the sympathetic neural influences, 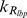 is a constant (tuned to reproduce the 2x greater increase in the lower body compared to the upper body during HUT), 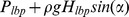 denotes the transmural pressure across the lower body arteries, 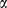 denotes the angle of tilting from the supine position, and 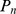 denotes the set point of the arterial baroreceptor.

Initial values of the reflex model parameters were given the same values as in [21]. The arterial baroreceptors were assumed to affect all the four effectors while the CP baroreceptors were assumed to affect only the peripheral resistances. Parameters that characterize the gains of the control of resistances, 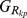, 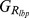 and 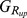, and unstressed volumes, 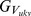, 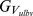 and 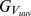 were given so that, at rest, the parallel arrangement of the segments provides the same overall response to that of the extrasplanchnic circulation in [20].

### Parameter estimation

Least square parameter estimation methods were then utilized to fit the parameters in the reflex model (Table 2) in order to best reproduce our experimental data and the published literature [3], [22]–[27] in a least squares sense. The model parameters were tuned to minimize the objective function, 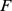, defined by
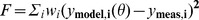
(8)where 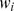 denotes weight corresponding to the 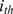 experimental dataset. 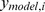 denotes the 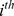 model output, while 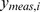 denotes the 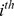 experimental measurement corresponding to the 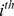 model output. Supine values of the mean systemic arterial pressure, mean cardiac output, mean stroke volume and mean heart rate from each individual subject, as well as their respective measured changes at 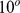, 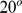, 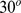, 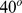, 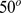, 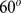 and 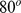 HUT were used in the model fitting process. In addition, the following constraints were also imposed: (i) the percent reduction in thoracic blood volume from supine to 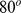 HUT was set in the range between 26 to 30% [3]; and (ii) the increase in lower body resistance from supine to 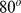 HUT was twice that of the upper body resistance.

Due to the limited experimental measurements as well as restrictions imposed by the model structure, we could not uniquely determine all reflex model parameters. Thus, we first performed sensitivity analysis to determine the effect of each model parameter on the model output. Each parameter, 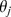, was perturbed from its baseline value by 10%, one at a time, and the change in the corresponding objective function was calculated. The dimensionless parameter sensitivity coefficient, 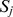, was evaluated using
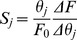
(9)where 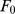 is the initial nominal value of the objective function corresponding to default model parameters.

Results of sensitivity analysis revealed that the most sensitive parameters consist of the static gain and baseline values of the individual effectors (i.e. venous unstressed volumes, heart period, vascular resistances and contractilities), which can be accounted for using physiological arguments. In particular, the baseline values of these effectors, e.g. 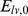 and 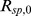 determine the supine values of the hemodynamic variables, while the static gains of these effectors, e.g. 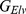 and 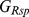 significantly affect the percentage changes of these variables in response to HUT. Parameters which mainly affect the transient response, for example those characterizing the time constants and latencies of the afferent and efferent pathways were found to have insignificant effects on the objective function.

Next, we carried out the optimization process on the remaining 27 parameters, and identified subset of dependent parameters from the covariance matrix upon completion of the optimization run. We then removed one dependent parameter which exhibited the highest covariance with any another model parameter (i.e. was co-dependent) and performed the optimization routine again. This process was repeated until we found a significant decrease in the objective value with further reduction in the number of parameters. Based on this procedure, nine model parameters were finally chosen to be optimized, while the remaining parameters were fixed to their original values (Table 2). Nonlinearly constrained gradient-based optimization method was used, with maximum and minimum bounds set for each parameter to ensure that the optimized values fall within physiologically reasonable limits. Mean and standard deviation values of the optimized parameters are listed in Table 2.

### Simulation protocols

The model was implemented using the Simulink toolbox in MATLAB (The Mathworks, Inc., Natick, MA, USA). In order to investigate the response of heart failure patients to HUT, we have simulated a heart failure scenario (NYHA Class III) by carefully modifying the parameters associated with heart failure to ensure that realistic simulation in terms of mean arterial pressure, mean left atrial pressure, mean pulmonary arterial pressure, mean central venous pressure, heart rate, stroke volume and cardiac output was achieved [28], [29]. These variations include a decrease in the cardiac contractilities (left ventricle, 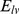: 80% decrease, and right ventricles, 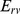: 40% decrease), an increase in the baseline pulmonary (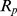: 40% increase) and systemic vascular resistance (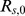: 25% increase) as well as total blood volume (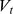: 600 mL increase), and desensitization of the reflex response (by modifying the static characteristics of the effectors [21], 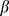 = 10).

The resulting simulated key haemodynamic variables at the supine position for both healthy and heart failure conditions are listed in Table 3, where the values are seen to agree with the published data. Since most papers do not limit their experiments to only one class of heart failure subjects, we have included all HUT studies performed on moderate to severe heart failure subjects (class II class III).

**Table 3:** Model simulated and published haemodynamic data for the healthy and heart failure subjects.

Variable	Healthy		Heart failure	
	Simulation	Experiment	Simulation	Experiment
Absolute value at supine position				
(mmHg)	88.4	74–97[1], [23], [28], [30], [31], [33]	87.9	86–103[28], [29], [31]–[34], [36], [41]
(mmHg)	6.7	6–10[1], [30], [31]	24.4	22–27[28], [29], [31], [36], [41]
(mmHg)	15.3	13–18[1], [30], [31]	34.1	34–38[31], [36], [41]
(mmHg)	4.4	3–6[1], [30], [31]	9.4	6–11[28], [29], [31], [36], [41]
(bpm)	68.8	53–74[1], [23], [28], [30], [31], [33]	82.3	74–88[28], [29], [31], [33], [34], [36], [41]
(mL)	69.1	55–112[1], [23], [30], [31], [33]	42.7	35–69[28], [29], [31], [33], [34], [36], [41]
(L.min-1)	4.84	4.0–6.4[1], [23], [30], [31], [33]	3.51	3.1–4.3[28], [29], [31], [33], [36], [41]
(mmHg.s.mL-1)	1.04	0.84–1.08[1], [30], [31]	1.34	1.09–1.57[28], [29], [31], [36], [41]
(mL)	122.7	82–132[1], [23], [33]	185.8	104–351[31], [33], [34], [36]
Changes from supine to HUT (Simulation: HUT)				
(mmHg)	4.1	HUT: 2–2.8[23], [34]	0.2	HUT: −1.4–0.6[28], [29], [34]
		HUT: 0–9[3], [24]		HUT: −28–0[31]–[33]
(%)	20.9	HUT: 10.6–19[23], [25], [34]	8.9	HUT: 0–10.8[28], [34]
		HUT: 15–55[3], [24], [30]		HUT: 0–1[31], [33]
(%)	−35.8	HUT: −29–34[23], [25], [34]	−10.9	HUT: −26.2[34]
		HUT: −30–45[3]		HUT: −7–15[31], [33]
(%)	−24.1	HUT: −19–18[23], [25]	−3.0	HUT: −14–8[28], [29]
		HUT: −30–15[3], [30]		HUT: −6–18[3], [24]
(%)	−20.2	HUT: −28[34]	−3.5	HUT: −13.3[34]
		HUT:-		HUT: ventricular end diastolic volume. Published 7[33]

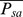
: mean arterial pressure; 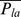: mean left atrial pressure; 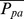: mean pulmonary arterial pressure; 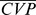: mean central venous pressure; 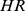: heart rate; 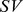: stroke volume; 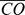: mean cardiac output; 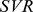: systemic vascular resistance; 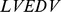: left ventricular end diastolic volume. Published range presented in the Table for the heart failure category was taken from experimental HUT studies performed on moderate to severe heart failure subjects.

## Results

### Comparison with experimental observations

**Figure 3:**
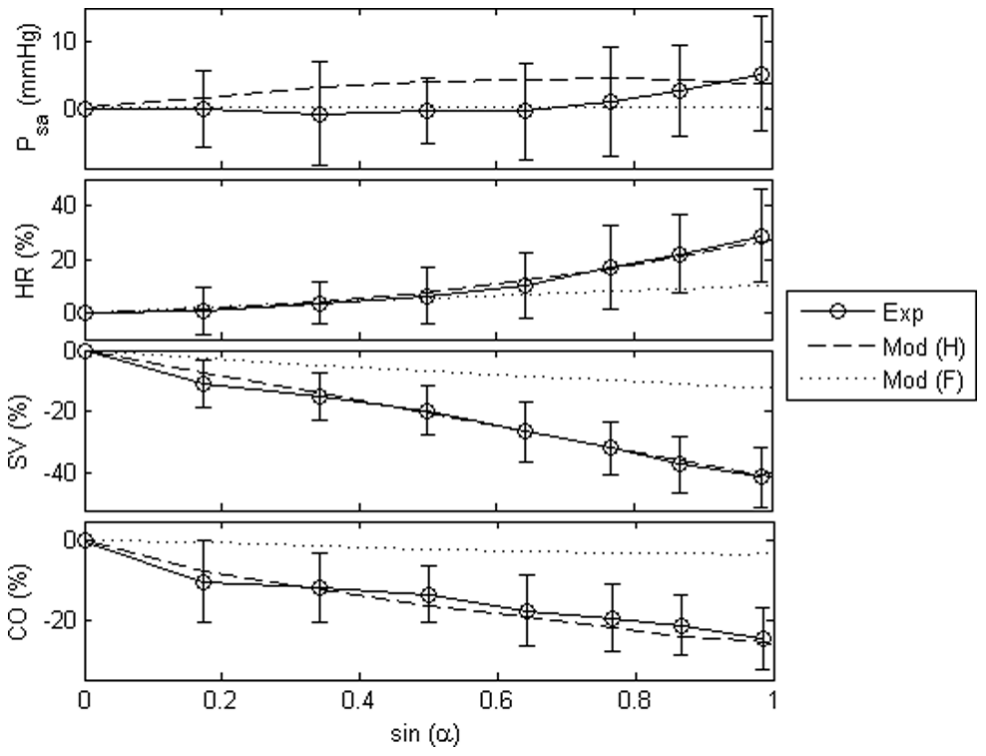
Changes in the mean arterial pressure (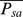), heart rate (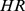), stroke volume (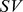), and mean cardiac output (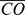). Absolute value change in the mean arterial pressure (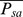), as well as percentage changes in the heart rate (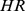), stroke volume (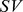), and mean cardiac output (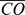) at varying levels of tilt angles (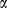) for both healthy and heart failure conditions. Exp: experimental measurements; Mod (H): model simulation results under healthy condition; Mod (F): model simulation results under heart failure condition.

To illustrate the trend of hemodynamic variables with increasing tilt angles under both healthy and heart failure conditions, steady state changes of key variables at varying levels of tilt angles from both the experiments and/or model were plotted in Fig. 3. Comparison between simulation results and published findings with regards to HUT response in both healthy [3], [23]–[25], [30] and heart failure subjects [28], [29], [31]–[34] were also presented in Table 3. There is a high degree of correlation between the model and experimental data in all variables, including absolute changes in the mean arterial pressure (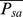), as well as percentage changes in the heart rate (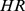), stroke volume (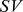), mean cardiac output (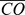) and LV end diastolic volume (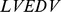). Compared to the healthy condition, heart failure scenario demonstrated a lesser degree of changes in all variables with progressive tilting.

Both simulated and experimentally measured mean arterial pressure remained relatively constant during graded HUT. Heart rate increased nonlinearly with progressive tilting (healthy: 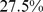; heart failure: 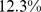 at simulated 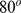 HUT condition), with a steeper increase at high tilt angles. On the contrary, cardiac output showed an opposite trend, where it decreased nonlinearly with increasing tilt angles, with a greater reduction at low tilt angles (healthy: 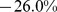 reduction; heart failure: 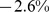 at simulated 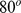 HUT condition). Stroke volume, on the other hand, decreased linearly with increasing tilt angles, with a 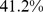 and 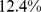 decrease respectively for the healthy and heart failure conditions at simulated 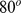 HUT condition.

In terms of regional blood volume, model simulations revealed a progressive blood volume shift from the thoracic/upper body compartment to the lower body compartment during HUT (Table 4). Thoracic blood volume (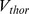), including volume in the pulmonary circulation and the heart chambers, as well as upper body volume (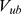) decreased nonlinearly with increasing tilt angle, with a decrease of −29.2% and −16.2% respectively at 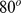 HUT for the healthy condition and −17.5% and −14.3% respectively for the heart failure condition. The percentage reduction in the central blood volume for the healthy condition in our simulation study (−29.2% at 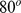 HUT, Table 4) was comparable to that reported by Smith *et al.* [3] (−26 to −30% at 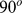 HUT). On the contrary, blood volume in the segment below the HIP, including the splanchnic (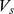), renal (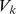) and lower body (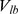) compartments increased nonlinearly with progressive tilting (12.1%, 21.1% and 25.5% respectively at 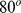 HUT for the healthy condition; 17.3%, 23.9% and 23.8% respectively for the heart failure condition).

**Table 4:** Simulated regional blood flow and volume for both healthy and heart failure conditions during supine and 70° HUT.

Variable	Healthy		Heart failure	
	0° HUT	80° HUT	0° HUT	80° HUT
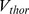 (mL)	954.6	675.6 (−29.2%)	1851.0	1526.7 (−17.5%)
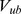 (mL)	453.2	379.6 (−16.2%)	414.2	354.8 (−14.3%)
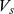 (mL)	1481.6	1659.2 (12.0%)	1417.1	1662.6 (17.3%)
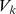 (mL)	375.5	455.0 (21.1%)	378.5	468.8 (23.9%)
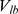 (mL)	817.6	1025.9 (25.5%)	779.2	964.4 (23.8%)
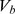 (mL)	324.8	154.5 (−52.4%)	352.9	179.2 (−49.2%)
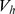 (mL)	121.0	116.2 (−3.9%)	131.7	125.7 (−4.6%)
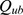 (L.min-1)	0.466	0.324 (−30.4%)	0.300	0.295 (−1.9%)
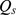 (L.min-1)	1.479	1.095 (−25.9%)	1.115	1.121 (0.5%)
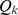 (L.min-1)	1.167	0.811 (−30.5%)	0.752	0.738 (−1.9%)
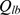 (L.min-1)	0.705	0.329 (−53.3%)	0.449	0.306 (−31.9%)
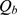 (L.min-1)	0.709	0.776 (9.5%)	0.672	0.699 (4.1%)
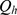 (L.min-1)	0.239	0.261 (9.5%)	0.226	0.235 (4.1%)

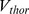
: volume in the thoracic region (including the pulmonary circulation and heart chambers); 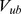 & 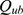: volume and flow in the upper body; 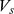 & 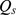: volume and flow in the splanchnic circulation; 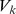 & 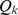: volume and in the renal circulation; 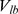 & 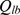: volume and flow in the lower body; 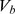 & 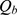: volume and flow in the cerebral circulation; 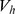 & 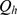: volume and flow in the coronary circulation.

On the other hand, regional blood flow in all compartments controlled by the reflex control mechanisms, i.e. upper body (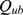), renal (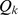), splanchnic (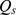) and lower body (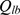), were shown to decrease nonlinearly with progressive HUT (Table 4). Cerebral (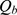) and coronary (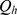) flow showed an opposite trend, with a 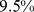 increase at 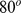 HUT for the healthy condition, and 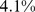 increase at 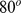 HUT for the heart failure condition.

### Comparison of healthy and heart failure response to HUT

Table 5 showed the effects of modifying individual factor associated with heart failure, including LV contractility (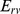), RV contractility (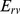), baseline systemic vascular resistance (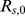), pulmonary vascular resistance (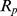), total blood volume (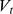), LV diastolic stiffness (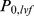) and desensitized reflex response (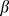), on key variables during supine and 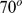 HUT, using model settings for the healthy scenario as baseline condition. Decreasing LV and RV contractility, as well as increasing LV diastolic stiffness decreased supine 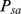, 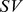 and 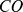, which resulted in an increase in 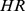 and 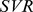. 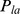, 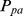 and 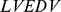 substantially increased with decreasing LV contractility, while less significant and opposite changes were observed with a decrease in RV contractility. Although reducing LV contractility and increasing LV diastolic stiffness simultaneously increased 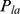, they produced directionally opposite changes in 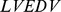. Increasing baseline systemic vascular resistance mainly increased 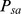 by increasing 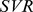, decreased 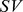, 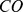 and 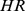. On the other hand, pulmonary vascular resistance had a minor effect on all variables, except for an increase in 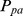. Total blood volume substantially increased all hemodynamic variables.

**Table 5:** Effect of individual parameter on key hemodynamic variables during supine and 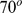 HUT.

Variable	Healthy	Heart failure	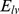	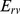	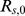	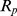	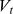	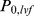	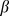
Absolute value at supine position									
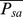 (mmHg)	88.40	87.90	76.98	87.33	91.56	87.72	91.20	85.09	88.40
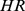 (bpm)	68.80	82.30	82.88	70.17	62.89	69.57	67.50	72.64	68.80
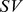 (mL)	69.10	42.70	48.32	67.41	67.41	67.49	86.97	60.76	69.10
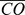 (L.min-1)	4.84	3.51	4.01	4.72	4.27	4.71	5.94	4.41	4.84
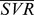 (mmHg.s.mL-1)	1.04	1.34	1.08	1.05	1.23	1.06	0.84	1.10	1.04
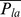 (mmHg)	6.70	24.40	11.31	6.62	6.60	6.54	11.39	9.65	6.70
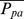 (mmHg)	15.30	34.10	18.27	15.13	14.10	18.30	22.35	17.49	15.30
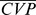 (mmHg)	4.40	9.40	4.80	4.56	4.17	4.39	8.43	4.21	4.40
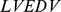 (mL)	122.70	185.80	155.49	120.27	123.32	120.62	141.71	112.21	122.70
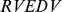 (mL)	94.69	88.72	73.87	99.28	92.54	95.99	118.05	87.68	94.69
Changes from supine to 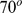 HUT									
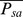 (mmHg)	4.10	0.20	6.32	4.78	3.40	3.98	12.08	4.99	−6.31
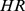 (%)	20.90	8.90	14.62	19.26	26.98	21.14	3.54	18.64	5.94
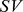 (%)	−35.80	−10.90	−29.76	−34.95	−36.19	−35.92	−14.62	−32.16	−30.16
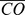 (%)	−24.10	−3.00	−19.88	−22.50	−19.84	−22.78	−10.57	−20.70	−25.71
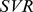 (%)	38.18	3.36	35.74	36.48	29.60	35.69	28.13	33.88	24.46
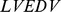 (%)	−20.20	−3.50	−13.83	−19.31	−20.61	−20.16	−5.82	−16.86	−19.49

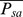
: mean arterial pressure; 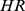: heart rate; 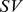: stroke volume; 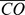: mean cardiac output; 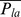: mean left atrial pressure; 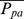: mean pulmonary arterial pressure; 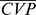: mean central venous pressure; 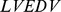: LV end diastolic volume; 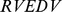: RV end diastolic volume; Columns from fourth to ninth refer to the simulation repeated (using healthy parameters) by individually modifying the following quantities to that used for the heart failure scenario: 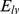: LV contractility; 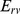: RV contractility; 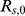: baseline systemic vascular resistance; 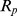: pulmonary vascular resistance; 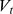: total blood volume; 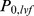: LV end diastolic stiffness; 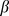: desensitized effector response.

With regards to the changes from supine to 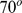 HUT, decreasing LV and RV contractility, as well as increasing total blood volume and LV diastolic stiffness produced directionally similar changes in 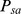 (larger increase), 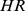 (smaller increase), 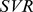 (smaller increase), 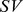 (smaller decrease), 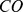 (smaller decrease) and 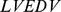 (smaller decrease). On the other hand, increasing baseline systemic vascular resistance lessen the increase in 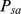 and the decrease in 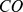 and 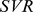 in response to 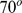 HUT, but amplify the increase in 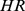. Desensitization of the reflex response lessen the degree of change in 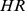, 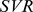 and 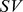 during HUT, resulting in a significant decrease in 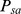 and a minor reduction in 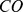. Out of all 7 simulated conditions, LV contractility, total blood volume and LV diastolic stiffness has more substantial effects on the response of 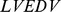 and 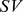 to 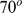 HUT. Among all hemodynamic variables, only 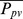 and 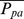 showed the same directional changes with regards to the response of 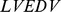 (percentage changes) to 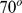 HUT in these conditions.

### Hypothesis testing

#### Effect of cardiopulmonary reflex

**Figure 4:**
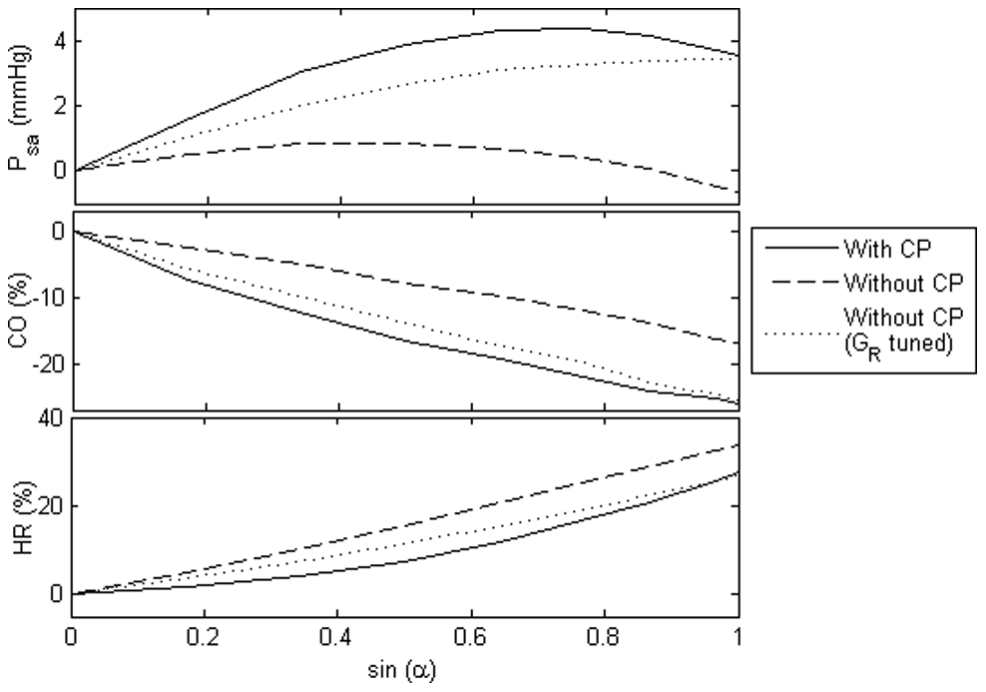
Effect of cardiopulmonary (CP) reflex on mean arterial pressure (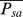), mean cardiac output (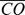), and heart rate (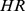). Absolute value change in the mean arterial pressure (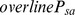), as well as percentage changes in the cardiac output (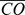) and heart rate (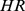), at varying levels of tilt angles (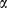) for the healthy condition. With CP: with CP reflex intact; Without CP: without CP reflex; Without CP (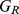 tuned): without CP reflex, but with all the four efferent resistance gains tuned to yield the same resistance values as that with CP reflex intact, at supine and 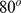 HUT.

To investigate the effect of CP reflex on graded HUT response, steady state response of the mean arterial pressure (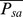), mean cardiac output (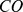) and heart rate (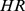) with graded HUT for three different conditions: (i) healthy condition with CP reflex intact, (ii) healthy condition without CP reflex (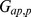  = 0), and (iii) healthy condition without CP reflex (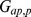  = 0), but with all the four efferent resistance gains tuned to yield the same resistance values as that with CP reflex intact, at supine and 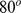 HUT, were plotted in Fig. 4. Without CP reflex, mean arterial pressure demonstrated a substantial fall with graded HUT. Mean cardiac output and heart rate showed opposite trends, where the absence of CP reflex diminished the decrease in mean cardiac output but amplified the increase in heart rate with progressive tilting. Although adjustment of the efferent resistance gains (with a multiplication factor of approximately 4 times each) yielded the same results as that with CP reflex intact at supine and 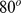 HUT, it substantially decreased the nonlinearity of the cardiac output and heart rate response to progressive tilting.

#### Effect of autoregulatory mechanism

**Figure 5:**
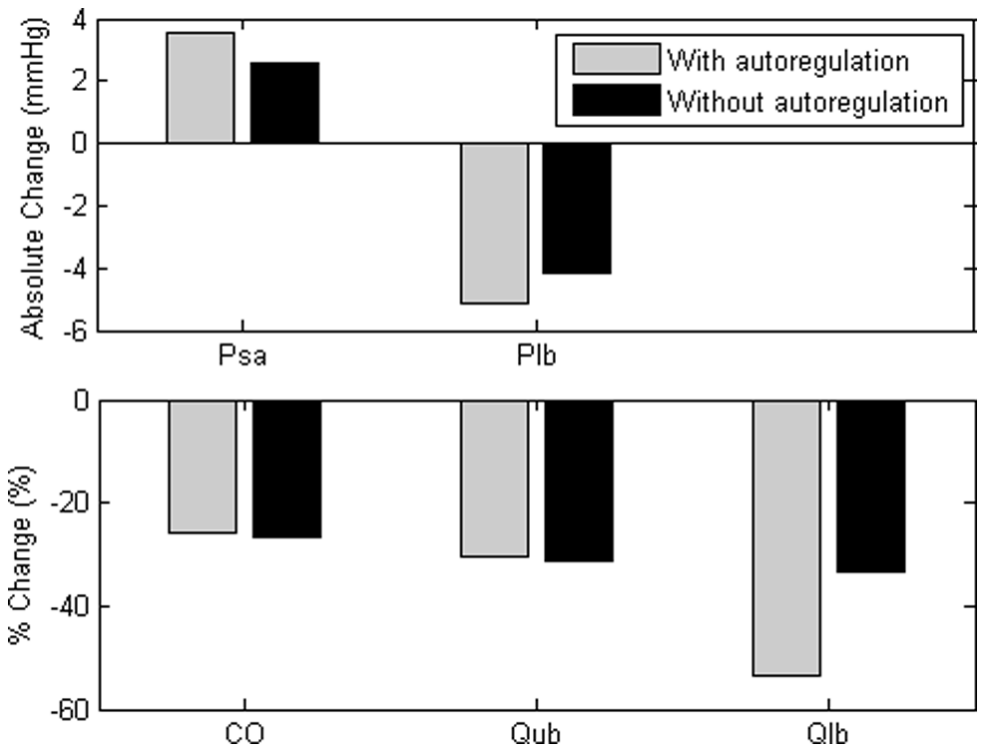
Effect of autoregulation on arterial pressure (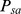), pressure in the lower body compartment (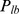), cardiac output (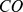) and flows in the upper and lower body compartments (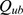 & 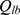). Absolute value changes in the arterial pressure (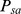) and pressure in the lower body compartment (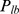), as well as percentage changes in the cardiac output (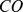), flows in the upper and lower body compartments (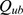 & 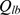) from the supine position to 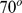 HUT for the healthy condition, with and without the autoregulatory mechanism.

Fig. 5 illustrates the effect of lower body autoregulatory mechanism on both global (arterial pressure & cardiac output) and regional (lower body pressure, upper body & lower body flow) hemodynamic response to 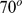 HUT in the healthy condition. Due to the combined effect of autoregulation with autonomic reflex control, flow in the lower body compartment (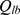) showed a 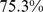 greater decrease as compared to that in the upper body compartment (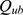) at 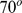 HUT (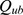: 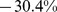; 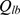: 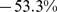 at 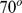 HUT). Meanwhile, with autoregulation, pressure in the lower body compartment (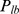) showed a greater decrease at 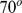 HUT. Despite causing a redistribution of blood flow among the various compartments, the autoregulatory mechanism did not significantly affect the global hemodynamic variables, with regards to the arterial pressure (with autoregulation: 3.6 mmHg; without autoregulation: 2.6 mmHg) and cardiac output (with autoregulation: −26.0%; without autoregulation: −26.8%).

#### Effect of diastolic ventricular interaction

**Figure 6:**
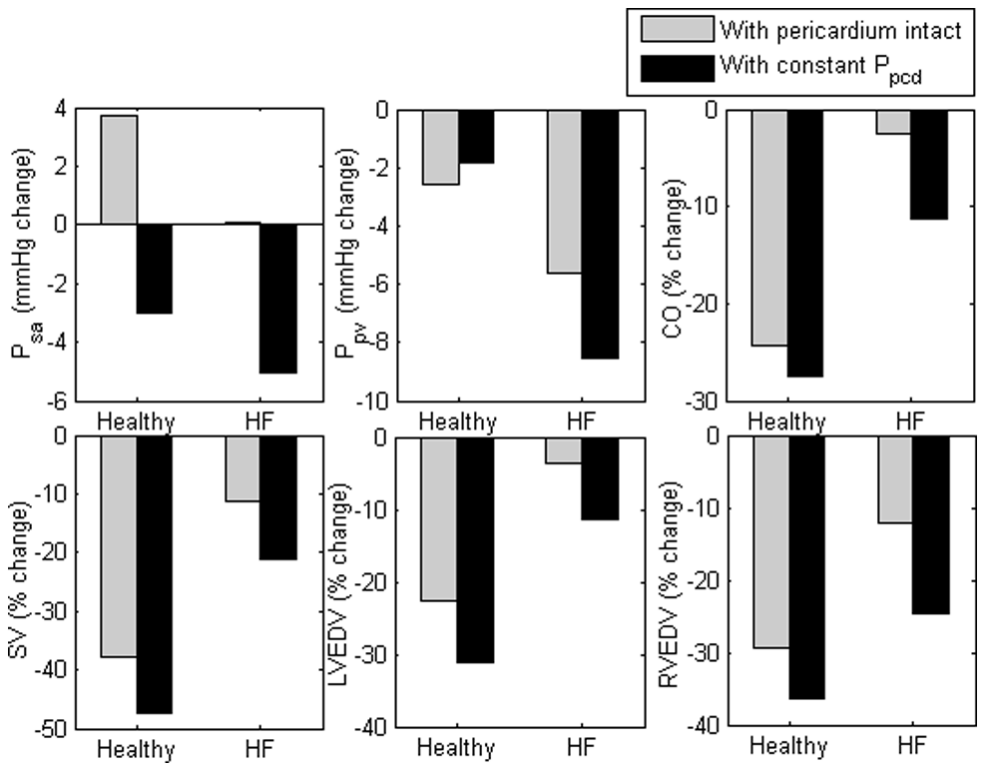
Effect of pericardial constraint on mean arterial pressure (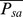), pulmonary venous pressure (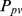), mean cardiac output (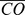), stroke volume (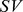), LV end diastolic volume (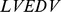) and RV end diastolic volume (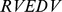). Absolute value changes in the mean arterial pressure (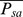) and mean pulmonary venous pressure (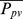), as well as percentage changes in the mean cardiac output (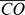), stroke volume (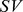), LV end diastolic volume (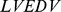) and RV end diastolic volume (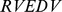) from the supine position to 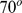 HUT for both healthy and heart failure (HF) conditions, with (i) pericardium intact and (ii) constant pericardial pressure (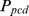).

Diastolic ventricular interaction refers to a situation in which changes in the function of one ventricle affects another ventricle, either through a series (i.e. through RV flow output) or direct (i.e. through the septum and pericardium) interaction [14]. To examine the effect of direct ventricular interaction (through the pericardium) on HUT response in both healthy and heart failure conditions, changes in the key hemodynamic variables from supine to 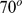 HUT with (i) pericardium intact and (ii) constant pericardial pressure (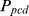, where 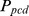 was held at its respective supine value) were plotted in Fig. 6. All variables, including mean arterial pressure (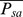), mean cardiac output (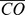), stroke volume (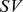), LV end diastolic volume (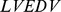) and RV end diastolic volume (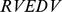), demonstrated a greater degree of reduction at 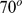 HUT when the pericardial pressure was held constant, except mean pulmonary venous pressure (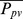 in the healthy condition. Compared to the healthy condition, the heart failure (HF) condition showed a larger difference between the two cases, i.e. with pericardium intact and with constant 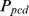, in all simulated variables except 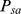 (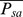:−6.7 mmHg (healthy) vs. −5.1 mmHg (HF); 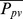: 0.77 mmHg (healthy) vs. −2.22 mmHg (HF); 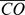: −13.3% (healthy) vs. −367.0% (HF); 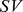: −24.6% (healthy) vs. −90.2% (HF); 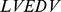: −37.0% (healthy) vs. −207.7% (HF); 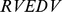: −23.4% (healthy) vs. −102.6% (HF)).

**Figure 7:**
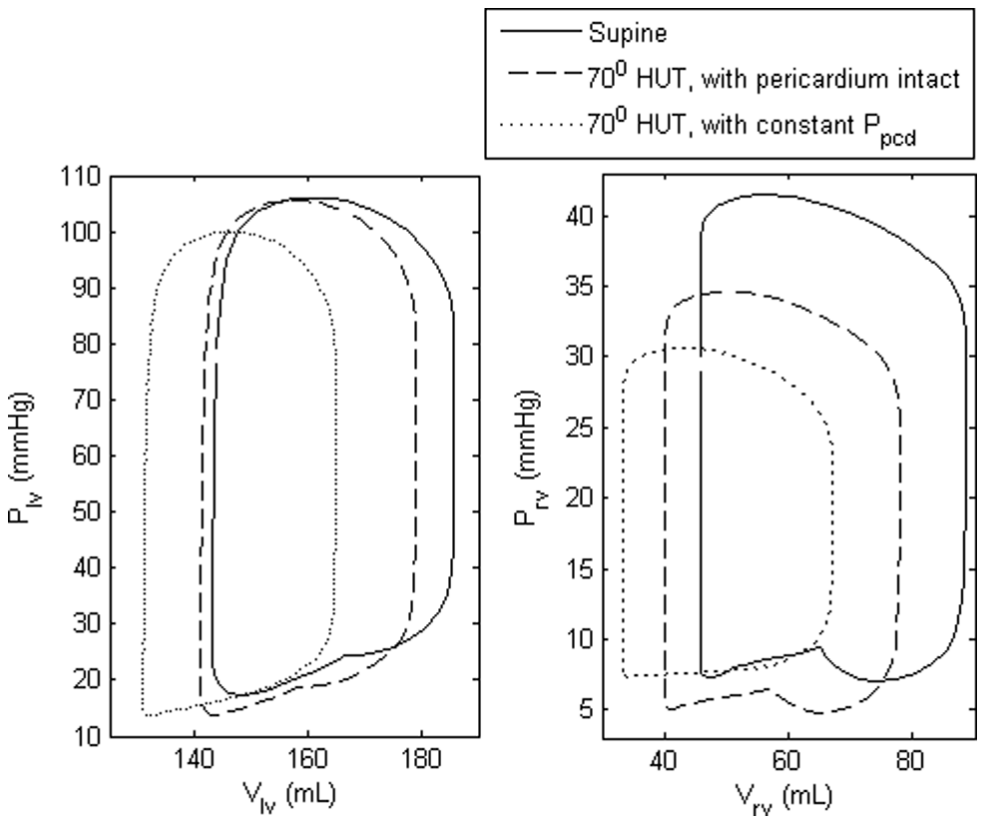
LV (left) and RV (right) pressure volume (PV) loops. LV and RV PV loops during supine and 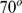 HUT for the heart failure condition, with (i) pericardium intact and (ii) constant pericardial pressure (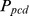).

As demonstrated in Fig. 7, a 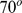 HUT in a heart failure subject with the pericardial pressure held constant produced a substantial decrease in both LV and RV end diastolic volumes. With the pericardium intact, the substantial decrease in the pericardial pressure surrounding the severely enlarged cardiac chambers with preload reduction (HUT) contributes substantially to a decrease in the elevated LV and RV filling pressures. This allows greater LV and RV filling, leading to a lesser decrease in the LV and RV end diastolic volumes with HUT and therefore stroke volume.

## Discussion

As observed by Taneja *et al.* [24], although both lower body negative pressure (LBNP) and HUT have been constantly used as models for hemorrhage and orthostatic stress due to their ability to produce central hypovolemia and comparable unloading of the cardiopulmonary and arterial baroreceptors, they produced directionally opposite effects on splanchnic volume due to their gravitational differences. Splanchnic circulation, which played an important role in preventing organ ischemia during a reduction in the central blood volume, was seen to fill during upright posture despite active vasoconstriction and venoconstriction (Table 4) [24].

Blood volume redistribution from the thoracic compartment to the lower portion of the body during HUT decreases central venous pressure, ventricular filling volume and subsequently ventricular pressure generating ability through the Frank-Starling relationship, leading to a decrease in the stroke volume (Fig. 3). Reduction in the central venous pressure, mean arterial pressure and pulse pressure with gradual HUT increases sympathetic activity through the cardiopulmonary and arterial baroreceptors, leading to an increase in the heart contractility, systemic vascular resistance, venous unstressed volume (venous tone) and heart rate. Our simulation results revealed that cardiopulmonary baroreceptors, which are sensitive to slight falls in preload or central venous pressure, contributes to the nonlinearity observed in the cardiac output response to progressive tilting (Fig. 4) through a substantial increase in the systemic vascular resistance at low tilt angles. This explains the experimental findings by Tuckman *et al.* [25], who found that the decrease in cardiac output in normal man tends to reach its maximum at 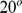 with less further decrease between 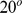 and 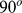. Meanwhile, heart rate, which is controlled mainly by the arterial baroreceptors, showed a steeper increase at higher tilt angles (Fig. 3 and 4), when preload change is sufficient to affect the mean systemic arterial pressure [35].

Numerous experimental studies [22], [23] have reported greater increases in the lower body vascular resistance compared to that of the upper body during HUT, and they attributed this to an interaction between the sympathetic nerve activity and the myogenic response in the lower extremities. Heusden *et. al.* [9] suggested that autoregulation and nonuniform resistance reflexes in the lower body might have considerable effect on venous return after HUT through a redistribution of flow. In the present study, we observed that while the local myogenic response affected flow redistribution from the lower body to other compartments in the body as that reported in the published experimental findings [22], [23], it did not substantially affect the global hemodynamic response to graded HUT (Fig. 5). Due to the nonlinearity in the pressure volume relationship, the compliance of the lower body compartment is significantly reduced at high transmural pressure occurring at high tilt angles, which subsequently limits an increase in the blood volume.

With regards to the response of heart failure subjects to progressive HUT, our simulation results showed that heart failure condition produced a blunted response to HUT as compared to the normal condition (Fig. 3), which is consistent with the published findings [28], [31]–[33]. Specifically, the increase in heart rate and systemic vascular resistance in response to HUT showed a substantial reduction, leading to a decrease in the systemic arterial pressure. On the other hand, as compared to the healthy condition, heart failure subjects showed a lesser decrease in LV end diastolic volume, and subsequently both stroke volume and cardiac output. A few mechanisms have been proposed to explain this: (i) beneficial effect of preload reduction on LV filling through direct ventricular interaction [34], [36]; (ii) blunted Frank-Starling response due to reduced LV distensibility [33]; and (iii) attenuation of effector response due to baroreflex desensitization [31].

By comparing the response of both healthy and heart failure subjects to graded HUT with and without pericardial constraint, we demonstrated the importance of direct diastolic ventricular interaction in heart failure subjects through the pericardium, which aids in lessening the changes in hemodynamic variables to upright posture (Figs. 6 and 7). The high pericardial pressure associated with severely enlarged cardiac chambers and elevated LV/RV filling pressure (mostly due to a reduction in LV contractility and an increase in blood volume) significantly decreased during HUT. This facilitates atrial emptying, yielding a much smaller decrease in the LV end diastolic volume, stroke volume and cardiac output as compared to a normal subject. Our simulation results is consistent with published findings by Pepi *et al.* [34], who proposed that gradual preload reduction enhances ventricular relaxation and diminishes the constraining effect of the pericardium.

With regards to the effect of LV distensibility on the response to upright posture, our model simulation results showed that increasing LV diastolic stiffness yielded a lesser degree of reduction in the LV end diastolic volume at 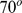 HUT (Table 5), despite diminishing the constraining effect of the pericardium through a decrease in both supine LV and RV end diastolic volumes. This explains the clinical findings by John *et al.* [33], who reported a blunted end diastolic volume (−7% at 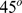 HUT), stroke volume (−7% at 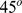 HUT) and cardiac output (−6% at 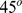 HUT) response to postural change in 48 heart failure patients with preserved LV ejection fraction, despite normal reflex response and decreased baseline end diastolic volumes. Furthermore, our simulation studies perturbing LV contractility, total circulatory volume and LV end diastolic volume revealed that the major determinant of the change in LV end diastolic volume to upright posture is baseline pulmonary venous pressure, which is consistent with experimental findings by Atherton *et al*. [36], who reported that only resting pulmonary capillary wedge pressure is predictive of the change in LV end diastolic volume during lower body suction. This can be explained by the fact that pulmonary venous pressure reflects both the size of the cardiac chamber as well as LV distensibility, which substantially affect the hemodynamic response to HUT.

It has been well established that congestive heart failure patients have markedly depressed baroreflex function [37]. By desensitizing the baroreflex function, we were able to simulate an attenuated response in the systemic vascular resistance, heart rate, cardiac contractilities and venous unstressed volume (i.e. the 4 effectors) to graded HUT in the heart failure subjects, as observed clinically [28], [29], [32]. As a result, systemic arterial pressure was decreased from the supine position to HUT. Interestingly, some severe heart failure patients showed a paradoxical vasodilation response to a reduction in the central blood volume [31], [32], as opposed to the normally expected vasoconstriction. Although various hypotheses, including diastolic ventricular interaction [36] and reflex sympathoinhibition [31], [32] has been proposed to explain this, the reasons remain unclear. Our simulation results showed that the inclusion of diastolic ventricular interaction and decreased baroreflex sensitivity in heart failure subjects produced an attenuated vasoconstriction response, but were unable to cause paradoxical vasodilation. Atherton *et al.* [38] suggested that the directionally opposite response observed in severe chronic heart failure patients to acute volume unloading may be caused by paradoxical activation of LV mechanoreceptors triggered by an increase in the LV end diastolic volume, which was not included in the present model.

Although various computational models have been developed to investigate the response to HUT, none of these studies have carefully looked into the changes in chronic heart failure patients. In the present study, we have analyzed the effects of individual factors associated with heart failure on the key hemodynamic variables, and showed the importance of diastolic ventricular interaction, baroreflex desensitization and blood volume on the blunted response observed in these patients. In particular, without the inclusion of diastolic ventricular interaction, the degree of change in LV end diastolic volume and cardiac output during HUT would be overestimated. The present model, in combination with a rotary blood pump model, could provide additional insights into the interaction between the cardiovascular system and the rotary blood pumps during a postural change at different pump operating points. Apart from that, being able to simulate the response of heart failure patients to HUT, it provides a platform for the evaluation of robust physiological pump control algorithms by easily allowing reproducible numerical experiments under identical conditions. Comparison among different controllers in maintaining both arterial pressure (to prevent hypotension) and left atrial pressure (to avoid pulmonary congestion and suction) during HUT can be done by carefully analyzing the simulation results.

## Study Limitations

Since the present study focused on the steady state response to graded HUT rather than transient changes, we have not taken into account the viscoelastic stress-relaxation properties of the systemic veins. Modeling the venous pressure volume dynamics using the viscoelastic property acts to reduce venous pooling during the first 30 s of HUT, thus producing little arterial blood pressure dip.

We have used a simplified representation of the CP reflex model in the present study, which was assumed to affect the systemic vascular resistance only. Conflicting experimental findings have been found in the literature with regards to the role of low pressure CP reflex on heart rate. Using both LBNP and neck suction experiments on healthy humans, Victor *et al.* [39] found that physiological variations in central venous pressure did not alter carotid baroreflex control of heart rate. To the contrary, using continuous recordings of RR interval, systolic arterial pressure variability and respiration, Lucini *et al.* [40] reported that heart rates are influenced by both carotid artery and CP reflexes. Due to limitation in data required to distinguish the effects of the two reflex mechanisms, we have chosen not to include the effect of CP reflex on heart rate. Despite that, our simulation results could reproduce the efferent response observed experimentally during graded HUT, with regards to systemic vascular resistance and heart rate.

## Conclusion

We have presented an optimized CVS model that is able to accurately reproduce experimentally measured steady state hemodynamic responses during graded HUT (from the supine position to 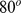), under both healthy and heart failure conditions. A number of features which provide important insights into the response of the CVS to upright posture have been included, such as diastolic ventricular interaction, a nonlinear PV relationship of the lower body compartment, arterial and cardiopulmonary baroreceptors, as well as autoregulatory mechanisms. Redistribution of blood volume from the upper body compartments to the lower portion of the body during HUT decreases LV end diastolic volume and pressure, stroke volume, cardiac output and pulse pressure, while maintaining mean arterial pressure. Compared to the normal condition, heart failure condition produced a blunted response to HUT with regards to the percentage changes in cardiac output, stroke volume, end diastolic volume and effector response with progressive tilting. This can be explained by a few mechanisms, i.e. (i) beneficial effect of preload reduction on LV filling through direct ventricular interaction; (ii) blunted Frank-Starling response due to reduced LV distensibility; and (iii) attenuation of effector response due to baroreflex desensitization.
